# Preliminary Monitoring and Observation of Fuel Cell Temperature Characteristics by Using NiCr-NiSi Thin-Film Thermocouple

**DOI:** 10.3390/mi16060639

**Published:** 2025-05-28

**Authors:** Zhihui Liu, Bohao Chang, Jinzhe Li, Yingyu Chen, Xingshu Wang, Zeren Rong, Zixi Wang, Wanyu Ding

**Affiliations:** 1 Guangdong Songshan Polytechnic, Shaoguan 512126, China; liuzhihui@mail.tsinghua.edu.cn (Z.L.); baobaohaohao@163.com (B.C.); 2 State Key Laboratory of Tribology in Advanced Equipment, Tsinghua University, Beijing 100084, China; 3 College of Aeronautical Engineering Institute, Civil Aviation University of China, Tianjin 300300, China; yingyu@163.com; 4 College of Electronic Information and Automation, Civil Aviation University of China, Tianjin 300300, China; lili@163.com; 5 College of Mechanical Engineering, Dalian Jiaotong University, Dalian 116028, China; dljtwxs@163.com; 6 College of Materials Science and Engineering, Dalian Jiaotong University, Dalian 116028, China; rongzeren2024@163.com

**Keywords:** NiCr-NiSi thin-film thermocouple, calibration, accuracy, thermoelectric effect, temperature measurement

## Abstract

This study presents the calibration methodology of NiCr-NiSi thin-film thermocouples and evaluates their application in real-time temperature monitoring and characterization of fuel cell thermal behavior. Experimental results reveal that the Seebeck coefficients of the NiCr-NiSi thin films remain stable after multiple calibration cycles, indicating good reliability and repeatability. Furthermore, the thermocouples demonstrate an ultrafast response time of less than 15 microseconds and reach thermal equilibrium within 200 microseconds under transient thermal inputs. These characteristics enable accurate and rapid temperature measurement of fuel cell plates up to 100 °C, which is critical for maintaining the safe and efficient operation of fuel cells.

## 1. Introduction

In recent years, Proton Exchange Membrane Fuel Cells (PEMFCs) have garnered significant attention in the energy sector due to their numerous advantages, including high energy conversion efficiency [1,2], low operating temperatures [3,4], rapid start-up capabilities, and zero pollutant emissions [3,5]. PEMFCs are capable of delivering efficient power output within environmentally friendly energy systems and are well-suited for applications such as portable electronic devices, electric vehicles, stationary power generation systems, and even aerospace technologies [6,7]. With the continuous advancement of PEMFC technologies in practical applications, ensuring their stable operation and optimizing performance have become critical areas of research focus [8,9].

During PEMFC operation, temperature plays a pivotal role in influencing the electrochemical reaction rates, membrane conductivity, water and thermal management, and overall system performance [10,11]. Operating temperatures that are either too high or too low may lead to membrane degradation, reduced catalyst activity, or reaction instability, thereby compromising the fuel cell’s lifespan and operational safety. Consequently, accurate and real-time monitoring of the temperature distribution within PEMFCs is essential for optimizing thermal management strategies, enhancing system reliability, and assessing safety margins.

At present, temperature monitoring in PEMFCs is typically performed through simulations or experimental measurements. Extensive simulation studies have been conducted to investigate PEMFC thermal behavior. For instance, Yang et al. [12] analyzed the effect of different heater temperatures on cell performance at an ambient temperature of 253.15 K using a numerical simulation based on a commonly used rectangular DC-channel fuel cell model. Yu et al. [13] developed a highly dynamic PEMFC stack model that considered the impact of temperature on performance and safety margins. Chugh et al. [14] employed a steady-state model in MATLAB, demonstrating that increases in operating temperature, pressure, and reactant humidity enhance PEMFC performance. However, in order to ensure numerical convergence, many simulation studies [15,16] simplify or neglect the influence of certain parameters, leading to deviations from experimental observations. To address this limitation, some researchers have implemented distributed in situ temperature measurements. For example, Ali et al. [17] embedded 16 T-type thermocouples into the anode and cathode flow plates of a high-temperature PEMFC based on polybenzimidazole. While effective, this approach disrupted the structural integrity of the fuel cell. In contrast, thin-film thermocouples offer a promising solution for in situ temperature measurement without damaging the PEMFC structure. Tang et al. [18] and Lin et al. [19] both fabricated T-type thin-film thermocouples using microelectromechanical systems (MEMS) to achieve real-time temperature monitoring within PEMFCs. Despite their effectiveness, MEMS-based thin-film thermocouples are associated with high fabrication costs. As an alternative, NiCr-NiSi thin-film thermocouples can be fabricated via magnetron sputtering, offering a more cost-effective solution. Furthermore, the high Seebeck coefficient of NiCr-NiSi materials makes them highly suitable for precise temperature measurements. These advantages highlight the significant potential of NiCr-NiSi thin-film thermocouples in PEMFC thermal diagnostics.

In this study, we present the in situ measurement of PEMFC operating temperature using magnetron-sputtered NiCr-NiSi thin-film thermocouples. The thermocouples were first fabricated using a magnetron sputtering technique and subsequently subjected to both static and dynamic calibration using laboratory instrumentation. Following calibration, the thermocouples were employed to monitor the temperature distribution during PEMFC operation, demonstrating their feasibility and effectiveness in real-world applications.

## 2. Laboratory Supplies and Equipment

**Figure 1:**
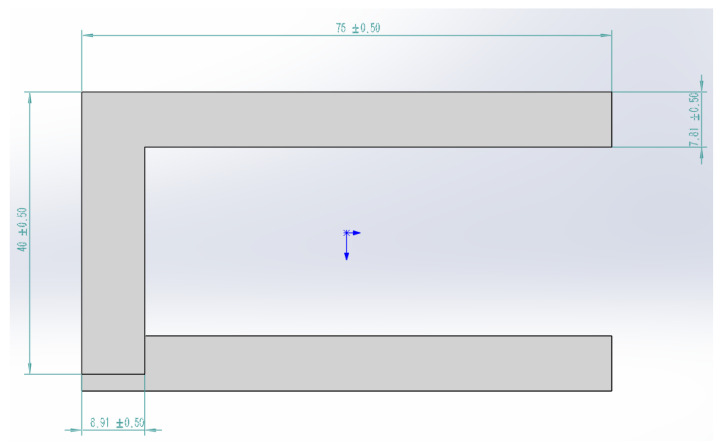
Diagram of a NiCr-NiSi thin-film thermocouple.

Figure 1 illustrates the structure of the NiCr-NiSi thin-film thermocouples employed in this study. These thermocouples are fabricated from high-purity nickel-chromium (NiCr) and nickel-silicon (NiSi) alloy films, materials that are well recognized for their excellent thermal stability and high sensitivity in temperature sensing applications, particularly under elevated temperature conditions. The thermocouple films possess a thickness of approximately 10 μm, which not only enhances sensitivity but also minimizes thermal mass—an important factor in achieving rapid thermal response.

The thin films are deposited onto a polyimide substrate using the magnetron sputtering technique. This method provides a uniform, adherent, and reliable coating, which is critical for long-term operational stability. Polyimide is selected as the substrate material owing to its outstanding thermal stability, mechanical flexibility, and resistance to high-temperature degradation, making it an ideal support layer for thin-film thermocouples in harsh environments.

The fabricated thermocouple exhibits the following geometric specifications: a width of 76 ± 0.50 mm, a height of 40 ± 0.50 mm, and a bottom thickness of 8.91 ± 0.50 mm. These precise dimensions are essential to ensure proper integration into the experimental system and to maintain uniform thermal contact and measurement reliability.

For calibration and experimental validation, high-precision instruments are employed to guarantee the accuracy and reproducibility of the temperature measurements. A thermostatic bath with a temperature control accuracy of ±0.1 °C provides stable thermal environments during calibration procedures. A high-precision thermometer, offering an accuracy of ±0.05 °C, is used for direct temperature readings. Additionally, a digital multimeter with a resolution of 0.01 mV is employed to record the thermoelectric voltage output of the thermocouples, facilitating precise voltage-to-temperature conversion.

This comprehensive setup, combining precision fabrication techniques with accurate temperature control and measurement instrumentation, ensures the high performance, sensitivity, and reliability of the NiCr-NiSi thin-film thermocouples throughout the experimental process.

Using magnetron sputtering, NiCr films, NiSi films, and their corresponding SiO_2_ protective layers were sequentially deposited. The NiCr and NiSi layers were each deposited to a thickness of 800 ± 50 nm, while the SiO_2_ protective layer was deposited to a thickness of 1000 ± 50 nm. All depositions were conducted in a vacuum chamber using high-purity (99.99%) targets: a NiCr target for the NiCr films, a NiSi target for the NiSi films, and a Si target for the SiO_2_ films.

Prior to film deposition, both the bipolar plates and mechanical masks underwent a standardized cleaning process consisting of sequential rinsing with acetone, ethanol, and ultrapure water, followed by drying in a dedicated drying chamber to ensure surface cleanliness. The deposition sequence began with the NiCr layer, followed by the deposition of the NiSi layer using a different mechanical mask, and finally, the SiO_2_ protective layer was deposited after replacing the mask once again. The specific deposition parameters for each film type are listed in Table 1 [20].

## 3. Static Calibration

Accurate temperature measurement is critical in both industrial and scientific contexts, particularly in processes that demand precise thermal control. To ensure measurement accuracy, static calibration of thermocouples over a defined temperature range is essential. This section describes the static calibration procedure performed over a temperature range spanning from room temperature to 150 °C—a range commonly encountered in manufacturing, materials testing, and other industrial applications.

Calibration was conducted at a series of predefined temperature points: 10 °C, 30 °C, 50 °C, 70 °C, 90 °C, 110 °C, 130 °C, and 150 °C. These points are uniformly distributed across the operating range to provide a representative characterization of the thermocouple’s thermal response. Such a distribution enhances the reliability of the calibration curve by capturing the sensor’s behavior under varying thermal conditions. While this range and point selection are sufficient for many practical applications, the calibration procedure can be readily adapted for scenarios requiring extended temperature limits beyond this standard interval.

### 3.1. Experimental Setup

The thermocouple under investigation, along with a calibrated standard thermometer, is immersed in a thermostatic bath capable of precisely controlling temperature. The bath is programmed to incrementally increase the temperature to each of the predefined calibration points. At each target temperature, the system is maintained for a minimum duration of 10 min to ensure thermal equilibrium is achieved and stable measurement conditions are established.

During this stabilization period, the readings from the standard thermometer and the thermoelectric voltage (thermopotential) generated by the thermocouple are recorded simultaneously. This procedure ensures that both instruments reach thermal and electrical stability, thereby minimizing potential errors caused by transient thermal fluctuations.

**Figure 2:**
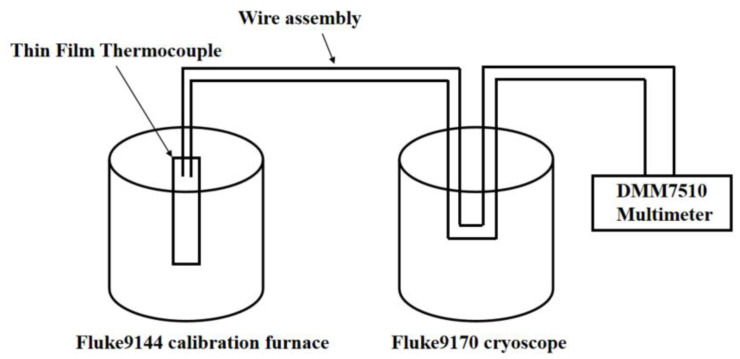
Static calibration flow chart.

The thermocouple’s output voltage at each temperature point is subsequently analyzed to establish a correlation between thermopotential and temperature. This data set is used to generate a calibration curve that enables accurate temperature conversion during actual measurements [21]. A schematic representation of the experimental setup is provided in Figure 2.

### 3.2. Data Processing

The raw data obtained from the calibration experiments were subjected to a rigorous preprocessing procedure to ensure data integrity and analytical reliability. Initially, all measured values were examined for the presence of outliers—data points that deviate markedly from the overall trend. Such anomalies may arise from thermal noise, transient instability of measurement instruments, or external environmental interferences. Outliers, by definition, do not reflect the intrinsic behavior of the system under study and may introduce significant bias into the results. Therefore, these anomalous values are identified and excluded through statistical or graphical methods to enhance the accuracy and robustness of the subsequent calibration analysis.

Following the outlier elimination, the next step involves computing the mean value of the remaining measurements for each discrete temperature point. Averaging reduces the impact of random fluctuations and minor systematic deviations, thereby yielding a more stable and representative dataset for curve fitting. This averaging process is essential for producing a smooth calibration response that closely reflects the true thermoelectric behavior of the thermocouple.

Subsequently, the averaged dataset was fitted to a polynomial function using the least squares regression method. This method is well-established in experimental data analysis, as it minimizes the cumulative squared deviation between the observed data points and the fitted curve. The polynomial fitting provides an optimal approximation of the thermocouple’s response characteristics, enabling precise interpolation and extrapolation within the calibrated temperature range. The derived calibration equation thus establishes a robust mathematical relationship between the measured thermoelectric voltage (thermopotential) and temperature, serving as the foundation for accurate and reproducible thermal measurements in future applications.

The general form of the calibration equation relating the temperature T and thermopotential V for a thermocouple is expressed as a polynomial function:

(1) $${V(T)=a}_{0}{\;+\;a}_{1}{T+a}_{2}T^{2}{\;+\;a}_{3}T^{3}+\cdot \cdot \cdot +a_{n}T^{n}$$

where a_0_, a_1_, a_2_, …, a_n_ are coefficients determined through the least squares fitting process, and T is the temperature.

### 3.3. Calibration Results

The experimental data obtained from the static calibration procedure were employed to construct the calibration curve for the NiCr-NiSi thin-film thermocouple. The results demonstrate that the thermocouple exhibits a high degree of linearity and excellent repeatability within the calibrated temperature range. This consistent behavior indicates that the thermocouple delivers reliable and predictable output, which is essential for high-precision temperature measurement applications.

To quantitatively evaluate the accuracy of the calibration curve, the measurement error at each temperature point is computed by comparing the thermocouple output to the reference thermometer readings. The error analysis reveals that, at the majority of the calibration points, the deviation is confined within ±0.5 °C, thereby confirming the high accuracy and reliability of the thermocouple. Furthermore, after conducting multiple independent experimental runs, the measured Seebeck coefficient—representing the thermoelectric sensitivity of the thermocouple—exhibits stable and consistent values. This further validates the thermocouple’s performance and underscores its suitability for repeatable thermal measurements.

**Figure 3:**
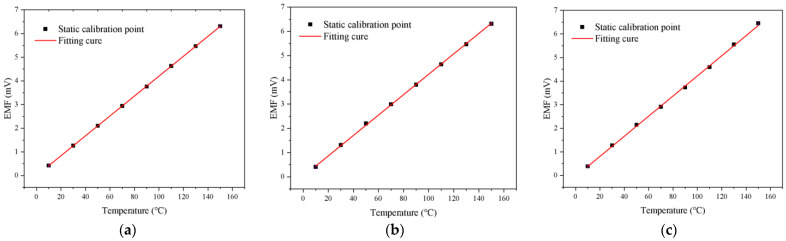
Temperature versus potential plot for three experiments. (**a**–**c**) Results of three sets of calibrations.

Figure 3 presents a series of calibration plots illustrating the thermoelectric response of the thermocouple over the temperature range of 10 °C to 150 °C. Subplots (a), (b), and (c) correspond to three separate experimental trials. In each graph, temperature is plotted along the *x*-axis and thermoelectric potential along the *y*-axis, providing a direct visual representation of the thermocouple’s voltage response to temperature changes.

Figure 3a shows the calibration data from the first experiment and reveals a near-linear relationship between temperature and thermoelectric voltage, consistent with the expected behavior of NiCr-NiSi thermocouples in this range. Figure 3b presents the results of the second trial, highlighting the reproducibility of the sensor’s performance across independent measurements. Figure 3c displays the third trial, further affirming the thermocouple’s stable response under identical conditions. Across the three experiments, the extracted Seebeck coefficient consistently converges to a value of approximately 42 μV/K, indicating the sensor’s high thermoelectric stability.

Together, these calibration plots provide compelling visual and quantitative evidence of the thermocouple’s linearity and repeatability. The close agreement across multiple experimental runs strongly supports the validity of the derived calibration curve and reinforces the thermocouple’s applicability for precise and stable temperature sensing in practical scenarios.

## 4. Dynamic Calibration

Dynamic calibration was carried out using a self-developed calibration system based on pulsed laser excitation. A Quantel Ultra 50 short-pulse infrared laser (wavelength: 1064 nm; pulse width: 7.6 ns; spot diameter: 2.56 mm) was employed to irradiate the center of the hot junction of the NiCr/NiSi thin-film thermocouple, with the beam incident normal to the surface of the thermocouple plane. To maintain a stable reference temperature at the cold junction, a Fluke 9170 ice point instrument was used, consistent with the static calibration setup [22].

**Figure 4:**
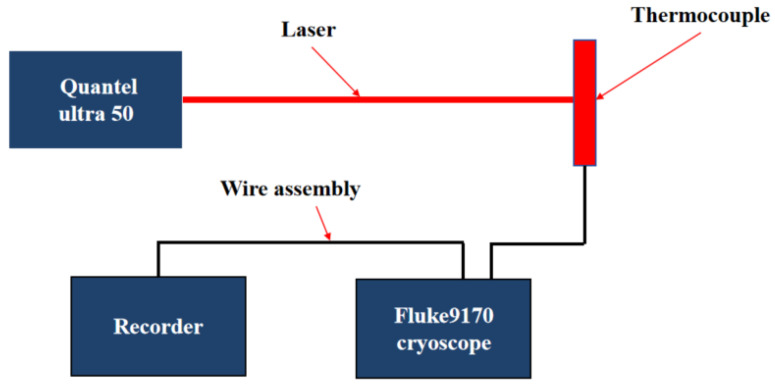
Dynamic calibration flow chart.

The thermoelectric voltage signal generated by the thermocouple was captured by an MR6000 data acquisition system through copper leads. This system provided a high temporal resolution of 5 ns and a voltage resolution of 65 μV, enabling accurate recording of the transient thermoelectric response during laser-induced heating. The specific experimental flow is shown in Figure 4.

**Figure 5:**
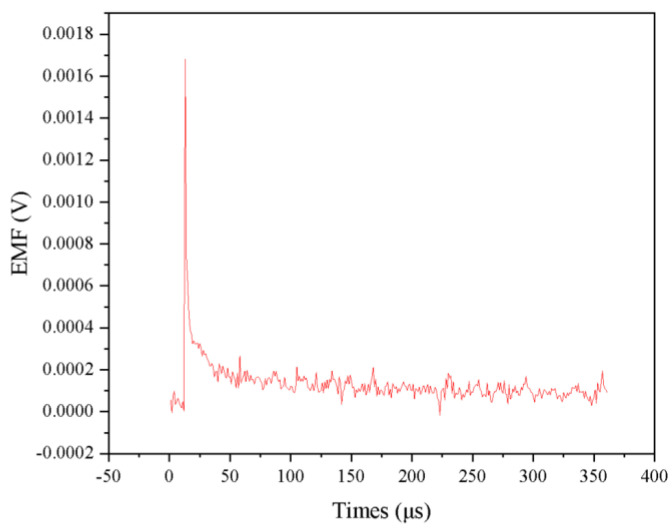
NiCr-NiSi thin-film thermocouple voltage versus time in dynamic calibration experiments.

Figure 5 illustrates the voltage–time response curves of the NiCr-NiSi thin-film thermocouple obtained during dynamic calibration experiments, showcasing its transient thermal response characteristics. As shown in the figure, the thermocouple rapidly produces a pronounced voltage peak of approximately 1756.9 μV immediately following the application of a transient thermal excitation. The emergence of this voltage peak clearly indicates that the NiCr-NiSi thin-film thermocouple possesses high thermal sensitivity and is capable of promptly and accurately detecting steep temperature gradients induced by rapid thermal transients. Such a rapid response is particularly critical for temperature monitoring in high-temperature environments characterized by abrupt and rapid thermal changes.

The voltage curve trend further reveals that, in addition to its fast initial response, the thermocouple exhibits a short thermal response time. Following the peak, the voltage signal declines sharply and stabilizes to thermal equilibrium within approximately 200 μs. This behavior reflects the thermocouple’s excellent thermal conductivity and its ability to dissipate heat efficiently, thereby enabling a swift return to a steady state. Such rapid recovery and smooth voltage decay underscore the thermocouple’s high thermal stability and its reliability in capturing accurate temperature signals under intense thermal perturbations.

The inset in the upper right corner of Figure 5 presents an enlarged view of the initial stage of the voltage–time curve, offering a more detailed depiction of the thermocouple’s rapid response to transient thermal input. As depicted, the voltage rises steeply within a very short time after the onset of thermal excitation, reaching a peak value of 1756.9 μV at approximately 1.7656 μs, as indicated by the red arrow. The fact that this peak is attained in under 2 μs highlights the thermocouple’s exceptionally fast thermal response time, confirming its capability to detect thermal transients on the sub-microsecond scale.

Immediately following the peak, the voltage signal undergoes a rapid decline, falling to approximately 500 μV within the next 10 μs, and subsequently approaches a stable level between 10 μs and 80 μs. This swift decay behavior indicates not only high thermal sensitivity but also efficient thermal diffusion, which prevents thermal lag and ensures timely heat dissipation.

Collectively, the dynamic response curves demonstrate that the NiCr-NiSi thin-film thermocouple features fast response, rapid recovery, and low signal fluctuation—attributes that confirm its outstanding performance in high-resolution, time-sensitive temperature measurements, particularly in transient and high-temperature environments.

Analysis of the dynamic calibration results presented in Figure 5 demonstrates that NiCr-NiSi thin-film thermocouples possess notable advantages for applications in high-temperature environments characterized by rapid thermal fluctuations. Their high sensitivity combined with rapid response times makes them particularly well-suited for precise temperature measurements in combustion processes and other fields involving transient thermal phenomena. Furthermore, the observed rapid voltage decay and stable steady-state response underscore the thermocouple’s capability to sustain reliable and accurate performance under severe thermal conditions.

## 5. Temperature Measurement of Fuel Cell Panel

**Figure 6:**
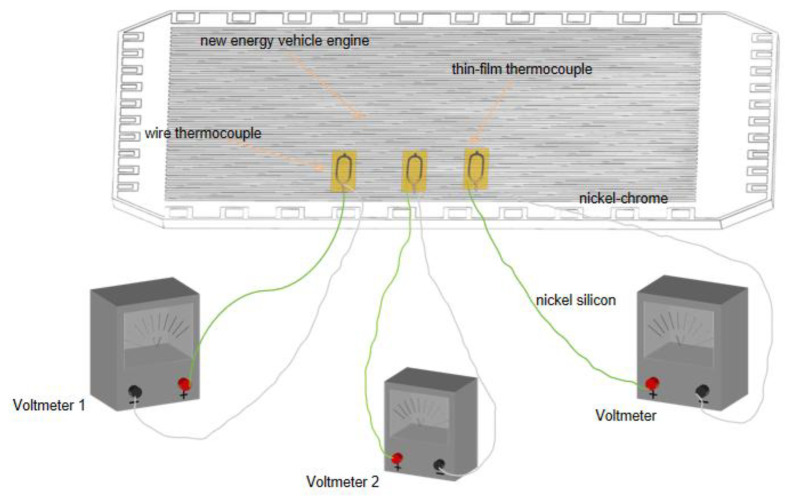
Fuel panel temperature connection diagram.

Figure 6 illustrates the temperature measurement setup of a fuel cell plate, where a NiCr-NiSi thin-film thermocouple is deposited onto the plate via magnetron sputtering to ensure intimate thermal contact. The temperature signals are acquired using a digital multimeter connected to the thin-film thermocouple through NiCr-NiSi wires, guaranteeing accurate and stable temperature readings.

**Figure 7:**
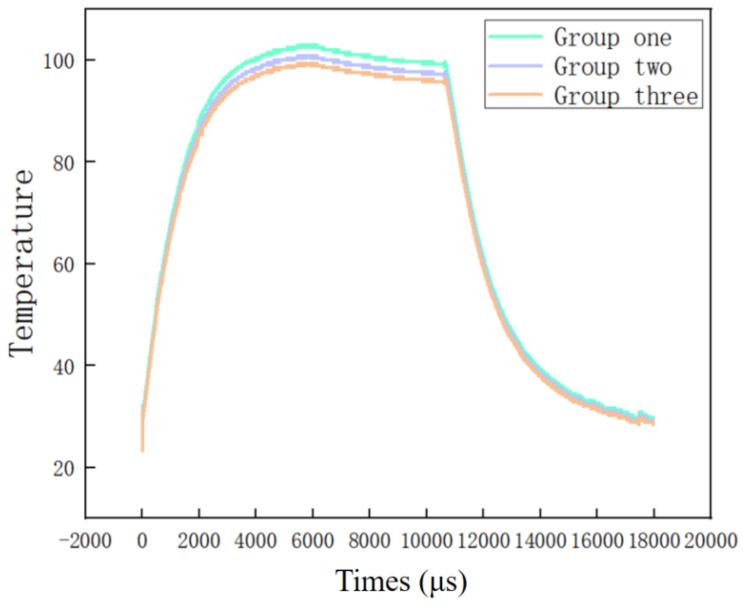
Thermal response curve of a new energy battery panel measured by NiCr-NiSi thermocouple across three experimental trials.

Figure 7 presents the temperature variation curves of the new energy cell plate recorded by the NiCr-NiSi thin-film thermocouples across three independent experimental runs. Each curve corresponds to a distinct trial, with time plotted along the horizontal axis and temperature along the vertical axis. The experimental results reveal a characteristic thermal response consisting of an initial rapid temperature rise, followed by a plateau phase, and concluding with a gradual temperature decline. Specifically, immediately after the experiment commences, the temperature rises sharply, reflecting the heat generation associated with the battery’s start-up process. Subsequently, the rate of temperature increase diminishes as the system reaches a quasi-steady state, likely indicative of thermal equilibrium or stable operating conditions of the battery plate. Finally, during the latter stage, the temperature gradually decreases, corresponding to heat dissipation following the shutdown of the thermopile, which halts continuous heat generation.

The data shown in Figure 7 exhibit high reproducibility, with each experimental run demonstrating a consistent thermal response under comparable conditions. Such reproducibility is crucial for validating the performance of the NiCr-NiSi thermocouple in precise temperature monitoring and attests to the sensor’s reliability and stability across varied test scenarios. The consistent thermal profile—characterized by an initial rapid increase, a stable plateau, and a gradual cooling phase—across all three trials confirms the stability of the experimental setup and supports the accurate characterization of the battery panel’s thermal behavior.

In the initial phase of the experiments, a sharp and rapid temperature increase is observed, commencing from approximately 20 °C and persisting for about 2000 microseconds (μs). During this interval, the temperature profile exhibits a steep gradient, indicative of a rapid heat accumulation process within the battery panel. This phenomenon is likely driven by the initiation of internal electrochemical reactions or exposure to external thermal stimuli. The thermal behavior during this transient phase is highly dynamic, characterized by rapid fluctuations that necessitate precise, high-resolution temperature measurements to accurately capture the evolving thermal state.

To meet this requirement, the NiCr-NiSi thin-film thermocouple demonstrates an exceptional response rate, effectively tracking these rapid thermal transients with high accuracy. Its fast response time enables real-time registration of abrupt temperature changes, ensuring critical data points are captured without loss. Such temporal resolution is essential for the accurate characterization of transient thermal phenomena, thereby providing valuable insights into the heat accumulation mechanisms and intrinsic thermal properties of the battery materials under investigation.

As the temperature approaches approximately 100 °C, the curve transitions into a plateau phase, maintaining relative stability for roughly 10,000 μs. This plateau corresponds to a state of thermal equilibrium wherein heat generation and dissipation reach a balance, resulting in a steady-state temperature. Throughout this phase, the NiCr-NiSi thin-film thermocouple continues to deliver stable and reliable temperature readings, highlighting its robustness and precision in sustained high-temperature conditions. The thermal equilibrium state during this period can be quantitatively described by:

Q_in_ = Q_out_ (2)

At thermal equilibrium, the rate of temperature change approaches zero (dT/dt = 0), and the system stabilizes at a constant temperature Teq. During this phase, the NiCr-NiSi thin-film thermocouple maintains stable and accurate temperature readings, thereby demonstrating its reliability and precision under sustained high-temperature conditions. The consistent performance of the thermocouple during thermal equilibrium is of particular importance for assessing the thermal stability and safety margins of the battery panel throughout its operational lifecycle.

Following this equilibrium phase, a gradual decline in temperature is observed between 12,000 and 20,000 μs, corresponding to the cooling stage of the battery panel. This temperature reduction is primarily attributed to the attenuation or complete removal of the external heat source, allowing the panel to dissipate the stored thermal energy into the surrounding environment.

Although minor variations are apparent in the cooling curves among the three experimental replicates, the overall cooling trends exhibit high consistency. Such reproducibility indicates that the NiCr-NiSi thin-film thermocouple preserves its measurement accuracy despite potential environmental fluctuations or instrument-related uncertainties. The observed deviations fall within acceptable experimental error margins, further validating the robustness and reliability of both the thermocouple and the employed experimental protocol.

## 6. Conclusions

This study presents a comprehensive investigation into the calibration and dynamic performance of NiCr-NiSi thin-film thermocouples, with particular emphasis on their application in monitoring the temperature characteristics of fuel cells. Experimental results demonstrate that these thermocouples possess outstanding transient response capabilities, generating a peak voltage of approximately 1756.9 µV within 15 microseconds, following transient thermal excitation and reaching thermal equilibrium within 200 microseconds. Such rapid response and high sensitivity are critical for accurately capturing the temperature fluctuations inherent to fuel cell operation.

Moreover, the Seebeck coefficients of the NiCr-NiSi thin-film thermocouples remained stable throughout multiple calibration cycles, underscoring their excellent thermal stability and measurement reliability. Static calibration tests further revealed that the thermocouples maintain a measurement error within ±0.5 °C across a temperature range spanning from ambient conditions up to 500 °C. This level of precision is indispensable for ensuring accurate and consistent temperature monitoring during prolonged fuel cell operation, especially under harsh, high-temperature conditions.

The applicability of NiCr-NiSi thin-film thermocouples for fuel cell temperature monitoring was further validated through experiments assessing the thermal response of a new energy battery panel. The thermocouples effectively captured rapid temperature changes, with a rise from approximately 20 °C to nearly 100 °C within 5000 microseconds, followed by a stable plateau phase and a gradual temperature decline. These thermal response patterns were consistently reproduced across multiple experimental trials, affirming the thermocouples’ capability to deliver precise and reliable temperature measurements under dynamic conditions.

In summary, NiCr-NiSi thin-film thermocouples exhibit exceptional transient response characteristics and robust thermal stability, rendering them highly suitable for applications demanding precise and rapid temperature measurements. Their demonstrated performance, combined with the potential for further optimization in material composition and structural design, positions these thermocouples as promising candidates for advanced temperature sensing in high-temperature and high-pressure fuel cell systems.

## Figures and Tables

**Table 1:** The deposition parameters for NiCr, NiSi, and SiO_2_ films.

Experimental Parameters	NiCr Film	NiSi Film	SiO_2_ Film
Target (wt.%)	Ni_90_Cr_10_	Ni_97_Si_3_	Si
Target purity	99.9%	99.9%	99.99%
Target base distance (mm)	120	120	120
Working gas	Ar	Ar	Ar/O_2_
Working pressure (Pa)	0.7	0.7	0.6
Flow rate (sccm)	20	20	20/10
Inversion time (μs)	1	1	1
Pulse frequency (kHz)	100	100	100
Sputtering power density (W/cm^2^)	1.90	1.90	3.33
Film thickness (nm)	800 ± 50	800 ± 50	1000 ± 50

## Data Availability

The original contributions presented in this study are included in the article. Further inquiries can be directed to the corresponding author.
